# Usability and Feasibility of a School-Based Digital Framework for Bullying Prevention

**DOI:** 10.3390/healthcare14030412

**Published:** 2026-02-06

**Authors:** Christopher Murray, Claudia G. Vincent, Dorothy L. Espelage, Luis Anunciacao, Hill Walker, Rita Svanks, Alberto Valido, Brion Marquez

**Affiliations:** 1 Center on Human Development, University of Oregon, Eugene, OR 97403, USAluisfca@gmail.com (L.A.);; 2 School of Education, The University of North Carolina at Chapel Hill, Chapel Hill, NC 27514, USAavalia@unc.edu (A.V.); 3 Department of Psychology, Pontifical Catholic University of Rio de Janeiro (PUC-Rio), Rio de Janeiro 22451-900, RJ, Brazil

**Keywords:** usability testing, digital intervention development, mental health, peer victimization, school-based prevention

## Abstract

**Highlights:**

**What are the main findings?**

- Students consistently showed strong acceptance and engagement with the technology-enabled framework.
- School personnel and parents/caregivers identified important implementation concerns, including alignment with school policies, appropriateness of language and materials, and the need for realistic, diverse, and high-quality video content to enhance student engagement.

**What are the implications of the main findings?**

- Technology-based reporting tools show promise for strengthening student voice in bullying prevention when aligned with school policies.
- Input from educators and families is essential to ensure materials are realistic, engaging, and contextually appropriate.

**Abstract:**

Bullying and school violence contribute directly to mental health difficulties among youth in the United States. **Background/Objectives**: This study describes the development and initial evaluation of a technology-enabled, multi-component school safety framework designed to support bullying prevention in middle and high schools. **Methods**: Students (*n* = 46), school personnel (*n* = 79), and parents/caregivers (*n* = 28) participated in three waves of usability and feasibility testing focused on a mobile application (Speak Out with Advocatr), companion classroom instructional materials, and guidelines for a school-wide safety campaign. Quantitative data were summarized using descriptive statistics and benchmark comparisons, and group differences across respondent roles were examined using analysis of variance with post hoc pairwise tests. Given small and unequal sample sizes, bootstrap resampling with 1000 resamples was used to obtain robust estimates of group means and confidence intervals. Qualitative responses were analyzed using content analysis. **Results**: Across waves, mean ratings generally met or exceeded predefined usability benchmarks, indicating favorable perceptions of the system. Findings indicated strong student acceptance and engagement with the framework. Adult participants expressed particular interest in restorative approaches to addressing student conflict, as well as concerns about preventing the recurrence of bullying behaviors. **Conclusions**: Findings provide initial support for the usability and feasibility of a multi-component, technology-enabled approach to school-based bullying prevention. Results also highlight the value of role-specific feedback for refining integrated mental health and safety interventions within school settings.

## 1. Introduction

Students’ emotional and physical well-being is fundamental to effective learning. Unfortunately, these aspects of health and safety are frequently jeopardized by peer victimization. Approximately one-quarter of children and adolescents are victims of bullying [1]. When such victimization persists over time, it can lead to serious consequences, including loneliness, depression, anxiety, suicidal ideation, and retaliatory violence that may threaten the safety of entire school communities [1,2]. Current approaches for keeping students emotionally and physically safe from bullying and school violence include school-based mental health programs [3], anti-bullying programs [4,5,6], and call-in tip lines [7,8].

Although well-intentioned, these approaches are not without limitations. Mental health programs often depend on specialized personnel and substantial funding, limiting scalability [9,10]. Many anti-bullying programs show limited effectiveness in rigorous evaluations [11,12,13], and anonymous tip lines may hinder effective follow-up due to the inability to clarify or contextualize reports. Because the frequency and persistence of peer victimization contribute to worse mental health outcomes [14], there remains a critical need to design prevention strategies that are affordable, actionable, context-sensitive, and serve to empower students to share safety concerns while concurrently equipping adults with information to quickly and effectively respond to potential safety concerns.

The growing integration of technology in educational settings has created new avenues for prevention and early intervention [15,16]. Mobile apps and digital tools can offer students discreet, accessible platforms to report health and safety concerns [17]. However, as such tools become more prevalent, so too does the need to assess their feasibility and usability given evidence regarding the operational and technical barriers associated with implementing such tools in school-based settings [17]. Usability and feasibility tests can provide insights into how different stakeholders (e.g., students, educators, and families) interact with such systems, and what adjustments might enhance engagement, comprehension, and adoption [15,16,18]. Such feedback is essential before proceeding to full-scale deployment.

The purpose of this study was to examine the usability and feasibility of SOARS (Student Ownership, Accountability, and Responsibility for School Safety), a technology-enabled, multi-component framework designed to support bullying prevention and school safety in middle and high school settings. Consistent with the program’s development focus, this study sought to gather role-specific feedback from students, school personnel, and caregivers to inform iterative refinement prior to large-scale implementation. This study addressed the following research questions:

RQ1: To what extent does the SOARS framework meet established usability benchmarks across students, school personnel, and caregivers?
RQ2: How do perceptions of usability and feasibility differ across stakeholder groups and across iterative testing waves?
RQ3: What qualitative feedback do stakeholders provide regarding strengths, concerns, and suggested improvements to the SOARS framework?
RQ4: What role-specific considerations emerge that may inform refinement and future implementation of technology-enabled bullying prevention systems in schools?

## 2. Materials and Methods

### 2.1. Participants

Participants were from two high schools, one in the Pacific Northwest and one in the Midwest. We conducted three waves of usability tests to maximize the opportunity to receive feedback from all users of the framework (i.e., students, school personnel, and parents). Students and parents participated in Wave 1 and Wave 3 testing and school personnel participated in all three waves (see Table 1).

In Wave 1, the student sample included 21 participants and was predominantly female (71.4%) and non-Hispanic or Latino (90.5%). Across Wave 1 and Wave 3, the majority of students were from grades 10 (38%) and 11 (38%) with fewer from grades 12 (21%), and 9 (2%).

At Wave 2, a total of 39 school personnel completed the study questionnaires. Demographic information was available for a subset of 20 participants. School personnel participants were primarily teachers (62%), including English/Language Arts, Health, French, Science, Math, History, Art, Media Skills, and special education. Educational Assistants comprised 27% of respondents and 9% of the school personnel respondents were school administrators.

The remaining school personnel included a test coordinator, a school counselor, a home interventionist, and a librarian. Seven parents participated in Wave 1 testing and 21 parents participated in Wave 3 testing. Wave 1 parent participants were from the Pacific Northwest site and included both male and female caregivers, with varied racial/ethnic backgrounds and educational levels.

### 2.2. Measures

User perceptions of the Advocatr system were assessed across three study waves using structured questionnaires tailored to each wave and respondent group. All items were rated on a 4-point Likert-type scale ranging from 4 (Strongly agree) to 1 (Strongly disagree). Two additional response options were available: Not applicable (NA), used when an item did not apply to the respondent’s role or experience, and No response (NR) when an item was skipped. Table 2 provides an overview of the domains assessed, respondent groups, and the number of items included in each wave. Measures evolved across waves to reflect the staged development of the Advocatr system and its associated instructional and campaign components.

In Wave 1, students, parents, and school personnel evaluated the system across four domains—Usability, Content, Implementation (school personnel only), and Video Scripts. Internal consistency reliability for Wave 1 measures was evaluated using Cronbach’s alpha across domains and respondents. Overall, the scales demonstrated acceptable to good internal consistency, including usability (α = 0.80), content (α = 0.90), implementation (α = 0.75), and video scripts (α = 0.78).

Wave 2 focused exclusively on school personnel and assessed Contextual Fit, Feasibility, and Relevance of instructional activities and informational briefs. Overall, internal consistency ranged from moderate to high, with stronger reliability observed for relevance–engagement measures (α = 0.88) and lower but acceptable reliability for instructional fit, feasibility, and relevance scales (α = 0.55–0.65).

In Wave 3, students, parents, and school personnel completed measures primarily assessing the ability to engage with campaign materials, along with clarity and relevance and contextual fit, depending on respondent group. Overall, internal consistency ranged from acceptable to good, with stronger reliability observed for informational brief and clarity measures (α = 0.76–0.82), acceptable reliability for safety items (α = 0.67), and lower reliability for engagement (α = 0.51).

In addition to the closed-ended items, all participants were given the opportunity to provide qualitative feedback through an open-ended question (“Please let us know any additional comments you might have”). School personnel were also asked to respond to an additional open-ended item (“As a staff member, I want this app to do…”), allowing them to suggest features or functions they considered important for school implementation.

### 2.3. SOARS and Advocatr Program Materials

The present project was developed to address ongoing challenges in identifying and responding to threats of violence within high school settings. The SOARS (Student Ownership, Accountability, and Responsibility for School Safety) framework targets both individual and ecological risk factors associated with violent behavior. At the individual level, risk factors include social isolation and heightened vulnerability to bullying based on gender, sexual orientation, disability status, race, a history of aggression, low academic performance, and mental health concerns [19,20,21]. These personal vulnerabilities often interact with ecological risks in families and schools. Family related risks include access to weapons, domestic violence, limited parental involvement, and conflictual parent–youth relationships. School-level risks encompass inadequate supervision, tolerance of bullying, and the normalization of harassment or sexual victimization. The interrelated nature of these factors complicates the design of effective interventions and often undermines school-based efforts to prevent violence [22].

**Figure 1:**
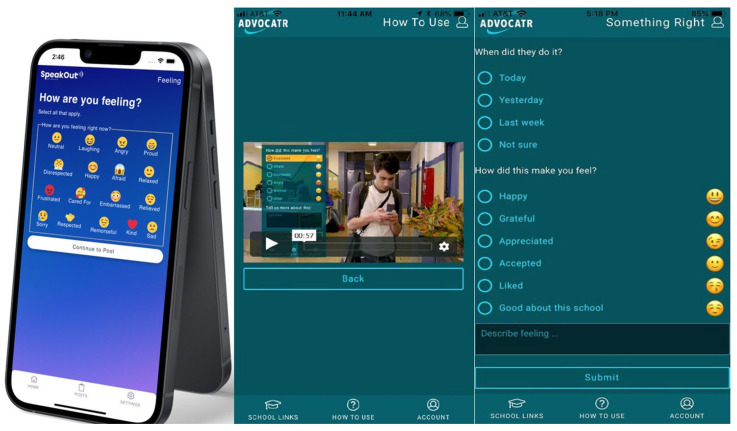
Advocatr App Screenshots.

The first component of SOARS is a reporting app called Advocatr, a mobile app and website (https://advocatr.org/) that allows students to report concerns about school safety quickly and confidentially. In Figure 1, we provide a screenshot of Advocatr’s three main functions: Report Something Wrong, Report Something Right, and Status of Reports. The Something Wrong and Something Right functions allow students to provide information about the event and the emotional impact it had on them. To facilitate confidence among students in the responsiveness of school staff to the report, the Status of Report allows students to see whether the report has been received, is in review, or is closed. Once entered, Advocatr reports go to a school-based SOARS coordinator who can reach out to the reporting student to address concerns or gather more information and then work within the school’s existing discipline policies to resolve the issue. Thus, Advocatr is confidential but not anonymous. To use the system, all students within a school are provided with a username and password and reports are then associated with that specific user.

**Figure 2:**
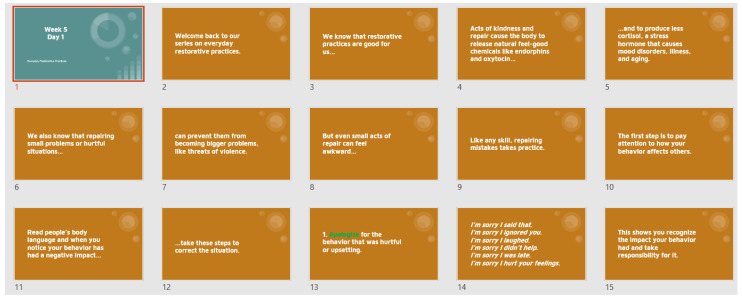
Excerpt from lesson plan on repairing relationships.

The second component of SOARS includes instructional activities delivered by teachers to support student use of the Advocatr app. These activities focus on (a) shared ownership of school safety, (b) self-advocacy, (c) physical and emotional safety, and (d) restorative practices and accountability. In Figure 2, we provide a sample lesson plan to illustrate how these modules are presented to teachers. Each activity is accompanied by a brief instructional video and a one-page “Did-You-Know” document which summarizes key information relevant to the target concept.

**Figure 3:**
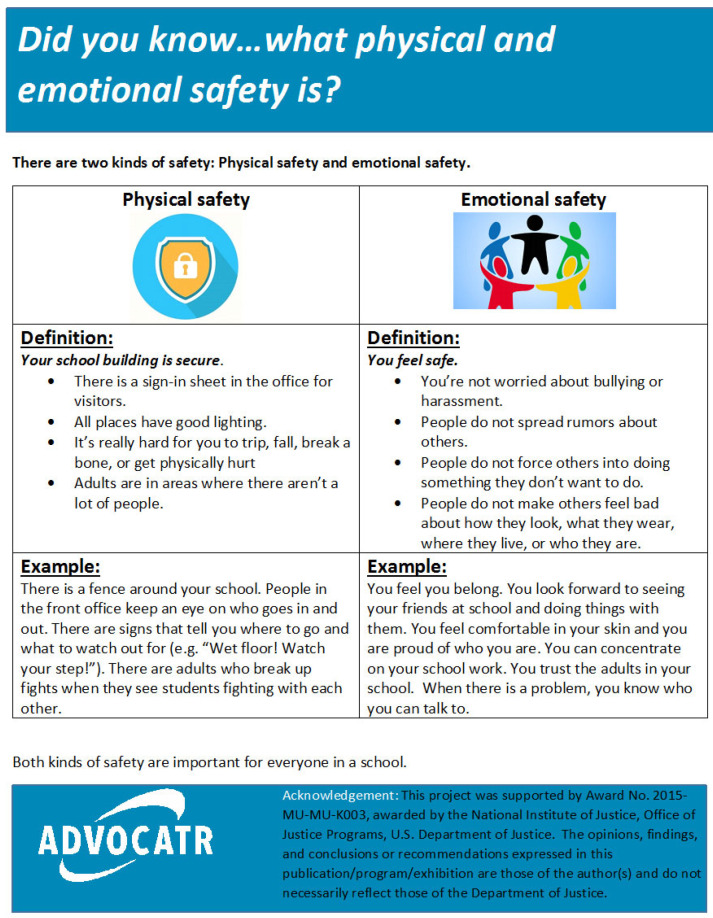
Sample Did-You-Know.

The third component includes informational briefs for teachers and parents. These briefs provide information about the research base supporting the framework and are designed to elicit teacher and caregiver awareness, knowledge, and motivation to support implementation. A sample brief is provided in Figure 3.

Although the SOARS framework primarily targets in-person bullying and school-based violence, its integration of digital tools for confidential reporting situates it within the broader ecosystem of online interactions that shape youth mental health. The Advocatr app functions as a technology-mediated communication platform in that it allows adolescents to share sensitive information, express emotional experiences, and seek adult support in digital spaces. In this way, SOARS intersects with contemporary concerns about cyberbullying and digital stress, offering an alternative model of constructive, prosocial technology use. By promoting psychological safety, emotional regulation, and trust in digital communication with adults, the framework contributes to the prevention of both online and offline victimization, supporting the mental health of youth in increasingly networked school environments.

### 2.4. Procedure

Recruitment for the usability tests was initiated by school administrators in collaboration with project staff. The project was approved by the home institution’s Internal Review Board and all participants provided informed consent prior to participating in the usability sessions. To assess multiple stakeholders’ perceptions at each wave, we conducted separate sessions for students, school personnel, and parents. Wave 1 solicited feedback on the Advocatr reporting tool and on the content of the initial scripts for the instructional videos. Wave 2 solicited feedback from school personnel about the usability of the instructional activities designed to support implementation of the Advocatr and accompanying one-page informational briefs. Wave 3 solicited feedback about initial videos, fully developed instructional materials (i.e., lesson plans), accompanying informational briefs, and components of the safety campaign materials.

Each feedback session lasted approximately two hours and were held at participating schools. During these sessions, the facilitator provided a brief introduction to the SOARS framework, its rationale, and key components. For example, during Wave 1, participants accessed the project test website on their smartphones or computers provided by the school. This website included the initial version of the Advocatr app reporting tool and all participants were guided through the app’s fields and functions. Once participants had reviewed the materials, they completed the usability survey. Similar procedures were applied to usability tests of other project materials (e.g., video scripts, videos, instructional materials, and safety campaign materials).

### 2.5. Statistical Analysis

Given the developmental and formative purpose of this study, hypotheses were not specified a priori. Instead, the study used usability and feasibility benchmarks and stakeholder comparisons to guide iterative program refinement. Descriptive analyses were first conducted to summarize user ratings across domains and respondent groups. Consistent with the formative purpose of the usability testing, benchmark scores were used as reference points, with mean ratings of 3.0 or higher on the 4-point scale interpreted as indicating acceptable usability. Negatively worded items were reverse scored to ensure consistent directionality across all measures.

To examine group differences across respondent roles (students, parents, and school personnel), one-way analyses of variance (ANOVA) were conducted for each domain when applicable. When overall effects were observed, post hoc pairwise comparisons with appropriate adjustments for multiple testing were performed. Given the small and unequal sample sizes across groups, bootstrap resampling with 1000 resamples were additionally used to obtain more robust estimates of group means and confidence intervals.

Qualitative data from open-ended responses were analyzed using content analysis. An initial topical indexing coding dictionary was developed to identify recurring themes, followed by categorization into broader thematic groups. Frequencies and proportions of responses within each theme were then calculated. Preliminary data processing and descriptive summaries were conducted in Excel (version 365; Microsoft Corporation, Redmond, WA, USA), whereas inferential statistical analyses were performed in Jupyter Notebook/Python (version 3.11; Wilmington, DE, USA) using Visual Studio Code (version 3; Microsoft Corporation, Redmond, WA, USA). Codes are available in the Supplementary File.

## 3. Results

### 3.1. Wave 1

Table 3 summarizes outcomes for the W1, W2, and W3 usability data. Across all three waves, students’ average scores were greater than the 3.0 benchmark on all usability tests except Wave 1, implementation of the Advocatr app (i.e., 2.56). Upon further examination of the raw data for this construct, it became clear that this lower rating was due to students’ lower ratings about whether using the app would fit with each school’s cell phone policy. During Wave 1, students also provided qualitative feedback about the Advocatr’s usability. The first theme focused on the incident reporting drop-down menu. Students suggested that the menu should allow multiple behaviors to be reported per event and two students suggested adding a “sexual harassment” option. The second theme focused on the video scripts and indicated that students’ felt that the scripts and forthcoming videos could be improved by making them more realistic. As one student stated, “Wording by the students in this script needs work to seem more genuine.” Another student noted:

“The scenes seem quite stereotypical. Students would most likely find the scenes cheesy and not be able to relate as much. I think the videos should show students who may try to lie or place blame on others and how the app could get to the truth because that totally happens”

Results of the Wave 1 ratings from school personnel indicated that ratings fell below the 3.0 benchmark, or between disagree and agree on the original response options for all four usability scales (usability, content, implementation, and video scripts). Qualitative data gathered from school personnel also suggested two themes. The first focused on adding additional behaviors to the drop-down menu (e.g., theft, sexual abuse, sexual assault, self-harm, stealing, and cyberbullying). The second theme focused on the importance of developing high quality videos. For example, one teacher suggested.

“These videos could be great or horrible depending on the quality. I think animations would be more relatable than bad acting.” Another remarked “Scripts previewed were of a commercial or PSA [public service announcement] nature, not tutorial”

Wave 1 parent/caregiver ratings of the Advocatr app usability and content both fell below the 3.0 benchmark but average ratings were above the benchmark for implementation and the video scripts. Qualitative data gathered from parents’ open-ended responses suggested two themes at Wave 1. First, several parents suggested adding additional options to the drop-down incident reporting menu, including sexual harassment, sexual misconduct, and stalking. Another theme focused on the “outcomes” of reporting and concerns about how the school administrators would respond to reports. As one parent stated, “……the school would take these reports seriously instead of allowing them to continue”

Wave 1 inter-factor correlations indicated a strong positive association between Content and Usability (r = 0.62, *p* < 0.001), suggesting substantial shared variance between these two dimensions. In contrast, correlations between Content and Implementation (r = 0.28, *p* = 0.09), Content and Video (r = 0.15, *p* = 0.39), Implementation and Usability (r = 0.15, *p* = 0.36), Implementation and Video (r = 0.05, *p* = 0.76), and Usability and Video (r = 0.20, *p* = 0.22) were not statistically significant. Overall, this pattern supports the relative distinctiveness of the Implementation and Video factors, while indicating partial overlap between Content and Usability.

Consistent with these descriptive patterns, inferential analyses at Wave 1 indicated significant differences by respondent role for Usability, Content, and Implementation. Students rated both Usability and Content significantly higher than school personnel, and higher than parents for Usability. Parents rated Implementation significantly higher than students. No significant group differences were observed for the Video Scripts scale, indicating broadly comparable perceptions of the video content across respondent groups at Wave 1.

**Table 3:** Means, Standard Deviations and Overview of Group Comparison Results.

	[1] Students		[2] School Personnel		[3] Parents		F	*p*	Post Hoc
*Wave 1*	M (SD)	95% CI	M (SD)	95% CI	M (SD)	95% CI			
Usability (app)	3.16 (0.70)	[3.05, 3.29]	2.85 (0.69)	[2.74, 2.98]	2.81 (0.68)	[2.62, 3.00]	8.10	0.001	1 > 2, 3
Content (app)	3.21 (0.62)	[3.11, 3.32]	2.88 (0.70)	[2.78, 2.99]	2.97 (0.67)	[2.83, 3.14]	9.42	0.001	1 > 2
Implementation (app)	2.56 (0.63)	[2.25, 2.88]	2.92 (0.61)	[2.78, 3.06]	3.25 (0.46)	[3.00, 3.50]	3.89	0.024	3 > 1
Video Scripts (video scripts)	3.03 (0.65)	[2.88, 3.17]	2.89 (0.72)	[2.74, 3.05]	3.05 (0.52)	[2.89, 3.21]	1.11	0.333	ns
***Wave 2***									
Contextual fit (Instruction)			3.24 (0.67)	[3.11, 3.37]					
Feasibility (Instruction)			3.03 (0.63)	[2.9, 3.16]					
Relevance (Instruction)			3.11 (0.54)	[2.96, 3.25]					
Relevance (Information briefs)			3.17 (0.38)	[3.0, 3.36]					
Ability to Engage (Information briefs)			2.84 (0.67)	[2.72, 2.95]					
***Wave 3***									
Ability to Engage (videos)	3.10 (0.76)	[3.0, 3.21]	2.96 (0.58)	[2.87, 3.05]	3.36 (0.76)	[3.22, 3.48]	11.4	0.001	3 > 2
Contextual fit (Instruction)	-	-	3.12 (0.61)	[3.03, 3.21]					
Ability to Engage (Information briefs)	3.26 (0.68)	[3.16, 3.35]	2.95 (0.58)	[2.87, 3.02]	3.16 (0.74)	[3.06, 3.26]	12.0	0.001	3 > 2; 1 > 2
Ability to Engage (Safety Campaign Video)	3.22 (0.63)	[3.12, 3.31]	3.06 (0.59)	[2.97, 3.15]	3.15 (0.72)	[3.02,3.28]	2.33	0.09	
Clarity and Relevance (Safety Campaign)	3.47 (0.55)	[3.39, 3.55]							
Contextual Fit (Safety Campaign) ^a^	-	-	3.23 (0.46)	[3.15, 3.3]	3.43 (0.73)	[3.3, 3.54]	7.28	0.01	3 > 2
Contextual fit (Lesson Plan, Safety Campaign)	-	-	2.96 (0.73)	[2.83, 3.08]					

Note. ns = not significant. ^a^ Results of an independent *t*-test.

### 3.2. Wave 2 Usability Tests

Wave 2 usability tests focused on school personnel perceptions of the instructional activities and informational briefs that were developed to support student use of the Advocatr app. First, ratings for contextual fit, feasibility, and relevance pertaining to the instructional activities were above the 3.0 benchmark. This pattern suggests that school personnel generally viewed the instructional activities as appropriate for their school context and feasible to implement within existing classroom routines. Consistent with these ratings, school personnel responses to open-ended response options were generally positive but provided important insights such as “It would be important to have all teachers do these activities around the same time” and “Overall I think these would be easy to implement into an extended homeroom……and I would explain some vocabulary words more in-depth…”

Second, school personnel rated the relevance of the informational brief above the 3.0 benchmark but average ratings for ability to engage students fell slightly below the benchmark (*M* = 2.84). This divergence suggests that although the content was perceived as meaningful, its current format may limit student engagement. Qualitative data gathered from open-ended responses suggested that school personnel did not think that the language used in the briefs was appropriate. As one respondent indicated “The reading level is wordy/difficult and needs to be short/easy/focused.” Another respondent stated “I think the info is good. Maybe better if a bit more succinct”

### 3.3. Wave 3 Usability Tests

Wave 3 focused on student, school personnel, parent/caregiver perceptions of components supporting student use of Advocatr. As shown in the bottom section of Table 3, student and parent/caregiver ratings of the videos, informational materials, and safety campaign materials were all above the 3.0 benchmark. However, school personnel ratings for the videos and the informational briefs fell below the 3.0 benchmark with regard to student engagement, as did their ratings on the contextual fit of the lesson plan for the safety campaign. This pattern mirrors Wave 2 findings, in which school personnel viewed materials as relevant but expressed concerns about their ability to engage students in practice. One participant’s comment captured this theme:

The second video….. was a little cheesy and neither one was diverse. Why kids ‘don’t report’ needs to be addressed and discussed with teachers? In videos, maybe express more positive outcomes of reporting. I would also show more screens of the app in the video —walk through the steps of making a report.

Another educator remarked:

Need a broader range of actors. The ones shown are stereotypically the ones that get picked on. Maybe adding an athlete or two and other groups would give more for those groups to respond and use the app without been ridiculed by the group.

Regarding the informational briefs, one educator said “Quick visuals are the best. If it’s too long students and staff alike may or may not read all of it”

## 4. Discussion

This study evaluated the usability and feasibility of the SOARS framework, a multi-component school-based violence prevention program designed to empower students, engage families, and support educators. Through three waves of usability testing, we sought feedback regarding the feasibility, clarity, and perceived value of SOARS components—including the Advocatr app, instructional materials, informational briefs, and safety campaign activities. Students consistently rated the core components of SOARS above the usability benchmark, suggesting strong potential for youth engagement. These findings support the framework’s emphasis on student voice and ownership in promoting school safety, as evidenced by the current literature [23,24]. Parents and caregivers also expressed generally positive views of the framework. In contrast, school personnel offered more critical perspectives—particularly regarding the app’s implementation feasibility, student engagement with materials, and the contextual fit of classroom-based components.

### 4.1. Empowering Student Voice Through Technology

A central goal of SOARS is to provide students with a safe, confidential way to report concerning behaviors and safety threats. The high usability and content ratings of the Advocatr app among students suggested that digital platforms can serve as effective tools for student-centered reporting. However, students’ concerns about whether the app aligned with existing school cell phone policies highlight a practical barrier to implementation. Such implementation challenges are common among new and emerging school-based mHealth efforts [17]. In the current project, student feedback prompted the development team to clarify that Advocatr is not intended for emergency use but rather for reporting low-level or persistent issues outside of class time. Importantly, students also advocated for expanding the app’s reporting categories to include sexual harassment and related misconduct. While the development team supported this change, implementation was hindered by school district concerns that were most likely due to liability or policy restrictions. This tension illustrates a broader challenge in school-based mHealth prevention and intervention efforts related to balancing student-driven needs with institutional constraints. Future iterations of SOARS must continue to negotiate these tensions and advocate for policy flexibility that aligns with student well-being.

### 4.2. Educator Concerns

In contrast to students, educator responses were more cautious. While they acknowledged the relevance of SOARS components, they raised concerns about their feasibility, the ability of materials to engage students, and the practicality of responding to potentially high volumes of Advocatr reports. These concerns reflect both the resource limitations many educators face and the importance of aligning new initiatives with existing instructional practices. Notably, school personnel ratings of the informational briefs and instructional videos fell below the engagement benchmark across waves. Their responses underscore that new safety technologies are more likely to be adopted when they are tightly integrated into existing routines (e.g., advisory periods, SEL lessons) and accompanied by clear protocols for responsibilities and role clarity [8,25].

Feedback from educators led to several design changes, including simplifying language, increasing the use of visuals, and updating video content to be more realistic and diverse. Although these revisions improved ratings in some areas over the course of the project, they did not fully address educator concerns as highlighted by the persistent challenge of developing materials that resonated equally with students, parents and educators. In response to these concerns, the development team refocused lesson content on everyday situations and actionable skills, and worked to align materials with broader frameworks such as Positive Behavioral Interventions and Supports (PBIS). These steps reflect the importance of contextual fit in usability and underscore the need to provide educators with both content and clear guidance on how to integrate innovations into their classroom routines.

### 4.3. Parent/Caregiver Perspectives

Parents and caregivers generally expressed support for the SOARS framework, particularly the concept of empowering students to speak up about safety concerns. Notably, results of our group comparisons indicated higher ratings from parents than educators on the engaging nature of the videos, the information briefs and contextual fit, whereas educators had lower ratings across all variables analyzed. However, they also voiced reservations about whether schools would respond adequately to student reports. This theme of trust, and whether reporting leads to meaningful outcomes, is critical because it suggests that transparency and accountability must be built into both the app and the broader framework to sustain family confidence and participation.

### 4.4. Implications

Findings from this study yield several implications for research, practice, and policy. First, the systematic incorporation of stakeholder feedback from students, educators, and caregivers provides formative data that can enhance contextual fit, optimize implementation feasibility, and improve the perceived relevance of program components when developing school-based digital health programs. Although common in the technology design literature, usability and feasibility testing remain underutilized in school-based prevention and mental health interventions [26,27]. However, when such data are gathered, significant implementation challenges often emerge [17]. Because such efforts are often resource intensive, gathering feedback from ultimate program end-users helps maximize feasibility, acceptability, and implementation fidelity prior to broader dissemination.

Second, results underscore the central importance of student voice in the design and sustainability of school safety and mental health frameworks. Students’ willingness to engage with the Advocatr app and their emphasis on content realism, inclusivity, and confidentiality suggest that youth participation is integral to the ecological validity of prevention tools. When students perceive interventions as authentic, responsive, and representative of their lived experiences, they are more likely to sustain engagement and internalize pro-social norms around reporting and safety.

Third, educator readiness and institutional alignment emerged as key determinants of implementation quality. Participants’ concerns regarding feasibility, workload, and the responsiveness of disciplinary systems highlight the necessity of embedding professional development and administrative infrastructure into the rollout of technology-enabled prevention programs. Building educators’ capacity to respond restoratively to address the emotional impact of behavior as well as rule violations may increase students’ trust in adult responsiveness and, consequently, their willingness to report concerns. This orientation shifts the focus from punitive discipline to relational repair and emotional regulation, aligning with broader frameworks such as Positive Behavioral Interventions and Supports [28,29].

Finally, structural and policy constraints, including restrictive cell-phone policies or limited reporting categories, can impede adoption and undermine credibility. These barriers illustrate the need for multi-level collaboration among developers, school leaders, and district policymakers to ensure that safety technologies are supported by coherent institutional policies and clear implementation protocols.

Future research should build on these findings by employing larger and more diverse samples to enhance generalizability and by examining the longitudinal relationship between perceived usability, sustained use, and student outcomes. Incorporating validated usability instruments and implementation science frameworks [30] will allow for a more systematic assessment of contextual factors influencing adoption, scalability, and effectiveness. Such efforts will be essential to advancing evidence-based, technology-supported approaches to promoting violence prevention and positive mental health outcomes among youth in school settings.

### 4.5. Limitations

Several limitations warrant further consideration when interpreting these findings. First, the study involved a relatively small and geographically limited sample drawn from two high schools, which constrains the generalizability of results. Future research should recruit larger and more diverse samples to examine whether patterns observed here replicate across school contexts, grade levels, and demographic subgroups. Second, the survey instruments were adapted from existing usability measures rather than being psychometrically validated for this specific context. As such, the internal consistency and construct validity of these measures remain to be established. Third, although participants provided rich feedback on the usability and perceived feasibility of SOARS components, the study did not assess subsequent behavioral outcomes such as actual adoption, sustained use, or downstream effects on school climate and violence prevention. Longitudinal research is needed to determine whether favorable perceptions of usability translate into meaningful behavioral and systemic changes. Finally, as with most early-stage development studies, participants’ responses may have been influenced by novelty effects or social desirability, which should be addressed in future iterations through independent replication and mixed-method triangulation.

## 5. Conclusions

This study provides initial support for the usability and feasibility of SOARS, a multi-component, technology-enabled framework for bullying prevention, with particularly strong acceptance observed among students across iterative testing waves. At the same time, adult stakeholders identified key implementation considerations, including alignment with school policies, the need for clear procedures for triaging and responding to reports, and preparation of staff to use restorative and supportive approaches when addressing student concerns. Findings also suggest that classroom materials and campaign media should be concise, visually engaging, and reflective of diverse student experiences to support educator uptake and sustained use. These results yield practical recommendations for schools considering similar approaches, including the importance of implementation planning that specifies reporting workflows, roles, and training supports alongside ongoing refinement of engagement-focused materials. Future research should examine implementation at scale and over time, including longitudinal assessment of system use, response processes, and downstream outcomes related to school climate, bullying involvement, and student mental health, as well as variability in effectiveness across school contexts.

## Figures and Tables

**Table 1:** Participants by Wave.

Participant	Wave 1 (N)	Wave 2 (N)	Wave 3 (N)
Students	21		25
School Personnel	17	39	23
Parents	7		21

**Table 2:** Overview of Usability and Engagement Measures Across Study Waves.

Wave	Respondent Group	Domain (Factor)	Items	Example Item
Wave 1	Students, Parents, School personnel	Usability	7	“The different functions of the app are easily accessible.”
Wave 1	Students, Parents, School personnel	Content	11	“The app makes it easy to report safety concerns.”
Wave 1	School personnel only	Implementation	5	“The app can be easily implemented in our school.”
Wave 1	Students, Parents, School personnel	Video scripts	6	“The videos present realistic scenarios.”
Wave 2	School personnel only	Contextual fit	6	“Activities fit with curriculum.”
Wave 2	School personnel only	Feasibility	6	“Easy to implement in classroom.”
Wave 2	School personnel only	Relevance	3	“Activities encourage students to use app.”
Wave 2	School personnel only	Relevance/Engagement	9	“Briefs are written in appropriate language.”
Wave 3	Students	Ability to engage	18–20	“Videos make me want to talk about school safety.”
Wave 3	Parents	Ability to engage	18–20	“My child could identify with actors.”
Wave 3	School personnel	Ability to engage	20+	“Videos support classroom activities.”
Wave 3	Students	Clarity and Relevance	7	I got a solid understanding of what this campaign is about.
Wave 3	School personnel	Contextual fit	7	“Campaign aligns with school values.”

## Data Availability

The data presented in this study are available on request from the corresponding author due to ethical and privacy restrictions associated with the use of sensitive participant data. However, the Python codes and Jupyter Notebook with the analytical procedures are available at https://osf.io/qpkfm/, last accessed on 2 February 2026.

## Supplementary Materials

The following supporting information can be downloaded at: https://www.mdpi.com/article/10.3390/healthcare14030412/s1. SOARS DP and DA.
