# Supplementary material for: Usability and Feasibility of a School-Based Digital Framework for Bullying Prevention
Source: Healthcare (Basel). 2026 Feb 6;14(3):412. doi: 10.3390/healthcare14030412 (PMC12897625; doi:10.3390/healthcare14030412)
Supplement: Supplementary file 1 [file healthcare-14-00412-s001.zip › healthcare-4077454-supplementary.pdf]

Supplementary Material: SOARS DP and DA

SOARS

>Note: > On January 30, 2026 > This notebook refers to the manuscript: > Usability of a School-Based Digital Framework for Bullying Prevention and Youth Mental Health Promotion > This notebook was last updated on January 30, 2026, to align all outputs with the manuscript. > After having the original data, running all cells will fully reproduce the results reported in the article. > For questions, please contact me at luisfca@gmail.com > Thanks

```
import pandas as pd
import numpy as np
from scipy import stats

import re
import statsmodels.api as sm
from statsmodels.formula.api import ols

import pingouin as pg

from itertools import product

import warnings
```

Data wave 1

```
df_wave1 = pd.read_csv(
    r"C:\Users\psipu\Dropbox\Luis\ai. Spanish Usability Paper\data_wave1_usability.csv",
    sep=";",
    encoding="latin1"
)
df_wave1
```

Clean

```
def clean_df(df):
    # standardize column names: lowercase, replace non-word chars with underscores, collapse multiple 
underscores
    df.columns = [
        re.sub("_+", "_", re.sub(r"[^\w]+", "_", c.strip().lower())).strip("_")
        for c in df.columns
    ]
    # drop rows that are entirely missing
    return df.dropna(how="all")

clean_df(df_wave1)
df_wave1.head()
```

Means and standard deviations table

```
summary = (
    df_wave1
    .filter(regex=r"^(usability|content|implementation|video)_")
    .replace({"na": np.nan, "nr": np.nan})
    .apply(lambda s: s.astype(str).str.replace(",", ".", regex=False))
    .apply(pd.to_numeric, errors="coerce")
    .assign(role=df_wave1["role"], id=df_wave1.index)  # CHANGED: add id to count unique participants
    .melt(id_vars=["id", "role"], var_name="variable", value_name="value")  # CHANGED: include id in melt
    .assign(factor=lambda d: d["variable"].str.split("_", n=1).str[0])
    .groupby(["factor", "role"])  # CHANGED: groupby on df (not Series) so we can aggregate id + value
    .agg(
        mean=("value", "mean"),  # CHANGED: specify column explicitly
        sd=("value", "std"),     # CHANGED: specify column explicitly
        n_participants=("id", lambda x: x[x.notna()].nunique())  # ADDED: valid participants
    )
    .reset_index()
)

summary.round(2)
```

```
df_wave1["role"].value_counts()
```

Cronbach's alpha

```
# Reliability table for wave 1
reliability_wave1 = []

factors_wave1 =
df_wave1.filter(regex=r"^(usability|content|implementation|video)_").columns.str.split("_",
n=1).str[0].unique()

for factor in factors_wave1:
    cols = df_wave1.filter(regex=rf"^{factor}_").columns.tolist()

    data = (
        df_wave1[cols]
        .replace({"na": np.nan, "nr": np.nan})
        .astype(str)
        .replace(",", ".", regex=True)
        .apply(pd.to_numeric, errors="coerce")
    )

    data = data.dropna(axis=1, how="all")
    data = data.loc[:, data.nunique(dropna=True) > 1]

    if data.shape[1] < 2:
        continue

    try:
        alpha, ci = pg.cronbach_alpha(data=data)
        reliability_wave1.append({
            "factor": factor,
            "n_items": data.shape[1],
            "cronbach_alpha": alpha
        })
    except:
        pass

reliability_wave1_df = pd.DataFrame(reliability_wave1)
print(reliability_wave1_df.round(3))
```

Correlation among factors

```
corr_data = (
    df_wave1
    .filter(regex=r"^(usability|content|implementation|video)_")
    .replace({"na": np.nan, "nr": np.nan})
    .apply(lambda s: s.astype(str).str.replace(",", ".", regex=False))
    .apply(pd.to_numeric, errors="coerce")
    .groupby(lambda c: c.split("_")[0], axis=1)
    .mean()
)

corr_results = pg.pairwise_corr(corr_data, method="pearson")
corr_results.round(2)
```

Bootstrap confidence intervals for mean differences

```
# Keep bootstrap exactly as before, but compute point means using the same cleaning as `summary`
np.random.seed(42)
bootstrap_ci = {}
point_means = {}

for f in ["usability", "content", "implementation", "video"]:
    # For bootstrap
    long_boot = (
        df_wave1
        .filter(regex=rf"^{f}_")
        .apply(pd.to_numeric, errors="coerce")
        .stack()
        .rename("value")
        .reset_index()
        .assign(role=lambda d: df_wave1.loc[d["level_0"], "role"].values)
    )

    # Point means matching `summary`
    long_clean = (
        df_wave1
        .filter(regex=rf"^{f}_")
        .replace({"na": np.nan, "nr": np.nan})
        .apply(lambda s: s.astype(str).str.replace(",", ".", regex=False))
        .apply(pd.to_numeric, errors="coerce")
        .stack()
        .rename("value")
        .reset_index()
        .assign(role=lambda d: df_wave1.loc[d["level_0"], "role"].values)
    )

    point_means[f] = long_clean.groupby("role")["value"].mean()

    diffs = []
    for _ in range(1000):
        with warnings.catch_warnings():
            warnings.simplefilter("ignore", FutureWarning)
            sample = long_boot.groupby("role", group_keys=False).apply(lambda x: x.sample(frac=1,
replace=True))
        diffs.append(sample.groupby("role")["value"].mean().to_dict())

    ci = pd.DataFrame(diffs).quantile([0.025, 0.975]).T
    ci.columns = ["ci_low", "ci_high"]

    bootstrap_ci[f] = (
        pd.DataFrame({"mean": point_means[f]})
        .join(ci)
        .reset_index()
        .rename(columns={"index": "role"})
    )

for factor in ["usability", "content", "implementation", "video"]:
    print(f"\n{factor.upper()} - Mean (matches summary) and Bootstrap 95% CI (unchanged):")
    print(bootstrap_ci[factor].round(2))
```

ANOVA

```
anova_results = {}

for f in ["usability", "content", "implementation", "video"]: # Loop over each predefined factor prefix
(hard-coded list)
    anova_results[f] = sm.stats.anova_lm(
        ols(
            "value ~ C(role)",
            data=(
                df_wave1
                .filter(regex=rf"^{f}_") # Select only columns whose names start with the current factor
prefix (e.g., "usability_")
                .apply(pd.to_numeric, errors="coerce")
                .stack()
                .rename("value")
                .reset_index()
                .assign(role=lambda d: df_wave1.loc[d["level_0"], "role"].values)
            )
        ).fit(),
        typ=3
    )

anova_table = (
    pd.concat(anova_results, names=["factor"])
      .loc[(slice(None), "C(role)"), ["df", "F", "PR(>F)"]]
      .reset_index(level=1, drop=True)
      .rename(columns={"df": "df", "PR(>F)": "p"})
      .assign(
          df=lambda d: d["df"].astype(int),
          F=lambda d: d["F"].round(2),
          p=lambda d: d["p"].apply(lambda x: "< .001" if x < 0.001 else f"{x:.3f}".lstrip("0"))
      )
)

anova_table
```

Post hoc

```
# Store post-hoc results
posthoc_results = {}

for f in ["usability", "content", "implementation"]:
    # Prepare long-format data (same as ANOVA prep)
    long_data = (
        df_wave1
        .filter(regex=rf"^{f}_")
        .apply(pd.to_numeric, errors="coerce")
        .stack()
        .rename("value")
        .reset_index()
        .assign(role=lambda d: df_wave1.loc[d["level_0"], "role"].values)
    )

    # Run pairwise t-tests with correction
    posthoc_results[f] = pg.pairwise_tests(
        data=long_data,
        dv="value",           # dependent variable
        between="role",       # grouping variable
        parametric=True,      # use t-tests (set False for Mann-Whitney)
        padjust="bonf",       # correction: 'bonf', 'fdr_bh', 'holm', etc.
        effsize="cohen"       # effect size
    )

# Display post-hoc for each factor
for factor, results in posthoc_results.items():
    print(f"\n{'='*60}")
    print(f"POST-HOC: {factor.upper()}")
    print('='*60)
    display(results[["A", "B", "T", "p-unc", "p-corr", "cohen"]])
```

Data Wave 3 Students

```
df_wave3_students = pd.read_csv(
    r"C:\Users\psipu\Dropbox\Luis\ai. Spanish Usability Paper\data_wave3_students.csv",
    sep=";",
    encoding="latin1"
)
df_wave3_students.head()
```

Clean

```
clean_df(df_wave3_students)
df_wave3_students
```

Means and standard deviations table

```
summary = (
    df_wave3_students
    .filter(regex=r"^(engage|infobrief|safety|clarity)_")
    .replace({"na": np.nan, "nr": np.nan})
    .replace(",", ".", regex=True)
    .apply(pd.to_numeric, errors="coerce")
    .assign(role=df_wave3_students["role"])  # Add the role column
    .melt(id_vars="role", var_name="variable", value_name="value")  # Reshape data
    .assign(factor=lambda d: d["variable"].str.split("_", n=1).str[0])
    .groupby(["factor", "role"])["value"]  # Group by both factor and role
    .agg(mean="mean", sd="std") #this is the sd of all results
    .reset_index()
)
summary.round(2)
```

```
summary = (
    df_wave3_students
    .filter(regex=r"^(engage|infobrief|safety|clarity)_")
    .apply(pd.to_numeric, errors="coerce")
    .groupby(lambda c: c.split("_", 1)[0], axis=1).mean()  # Person-level means
    .assign(role=df_wave3_students["role"])
    .melt(id_vars="role", var_name="factor", value_name="value")
    .groupby(["factor", "role"])["value"]
    .agg(mean="mean", sd="std") #here is the SD of all individual means (I don't want that)
    .reset_index()
    .round(2)
)

summary.round(2)
```

```
df_wave3_students["role"].value_counts()
```

Cronbach's alpha function reused from above

```
 # Reliability table for wave 1
reliability_wave1 = []

factors_wave1 =
df_wave1.filter(regex=r"^(usability|content|implementation|video)_").columns.str.split("_",
n=1).str[0].unique()

for factor in factors_wave1:
    cols = df_wave1.filter(regex=rf"^{factor}_").columns.tolist()

    data = (
        df_wave1[cols]
        .replace({"na": np.nan, "nr": np.nan})
        .astype(str)
        .replace(",", ".", regex=True)
        .apply(pd.to_numeric, errors="coerce")
    )

    data = data.dropna(axis=1, how="all")
    data = data.loc[:, data.nunique(dropna=True) > 1]

    if data.shape[1] < 2:
        continue

    try:
        alpha, ci = pg.cronbach_alpha(data=data)
        reliability_wave1.append({
            "factor": factor,
            "n_items": data.shape[1],
            "cronbach_alpha": alpha
        })
    except:
        pass

reliability_wave1_df = pd.DataFrame(reliability_wave1)
print(reliability_wave1_df.round(3))
```

Bootstrap confidence intervals for mean differencesm

```
from scipy import stats
import numpy as np
import warnings

# Bootstrap confidence intervals for means
np.random.seed(42)
bootstrap_ci = {}

for f in ["engage", "infobrief", "safety", "clarity"]:
    long_data = (
        df_wave3_students
        .filter(regex=rf"^{f}_")
        .apply(pd.to_numeric, errors="coerce")
        .assign(role=df_wave3_students["role"])  # Add the role column
        .melt(id_vars="role", var_name="variable", value_name="value")  # Reshape data
    )

    # Bootstrap means (1000 iterations)
    diffs = []
    for _ in range(1000):
        with warnings.catch_warnings():
            warnings.simplefilter("ignore", FutureWarning)
            sample = long_data.groupby('role', group_keys=False).apply(lambda x: x.sample(frac=1, 
replace=True))
        means = sample.groupby('role')['value'].mean()
        diffs.append(means.to_dict())

    # Get 95% CI for each group
    bootstrap_ci[f] = pd.DataFrame(diffs).quantile([0.025, 0.975]).T

# Display bootstrap CIs
for factor in ["engage", "infobrief", "safety", "clarity"]:
    print(f"\n{factor.upper()} - Bootstrap 95% CI:")
    print(bootstrap_ci[factor].round(2))
```

Data wave 3 parents

```
df_wave3_parents = pd.read_csv(
    r"C:\Users\psipu\Dropbox\Luis\ai. Spanish Usability Paper\data_wave3_parents.csv",
    sep=";",
    encoding="latin1"
)
df_wave3_parents
```

Clean

```
clean_df(df_wave3_parents)
df_wave3_parents.head()
```

Means and standard deviations table

```
summary = (
    df_wave3_parents
    .filter(regex=r"^(engage|infobrief|safety|safetycampaign)_")
    .apply(pd.to_numeric, errors="coerce")
    .assign(role=df_wave3_parents["role"])  # Add the role column
    .melt(id_vars="role", var_name="variable", value_name="value")  # Reshape data
    .assign(factor=lambda d: d["variable"].str.split("_", n=1).str[0])
    .groupby(["factor", "role"])["value"]  # Group by both factor and role
    .agg(mean="mean", sd="std") #this is the sd of all results
    .reset_index()
)
summary.round(2)
```

```
df_wave3_parents["role"].value_counts()
```

```
from scipy import stats
import numpy as np
import warnings

# Bootstrap confidence intervals for means
np.random.seed(42)
bootstrap_ci = {}

for f in ["engage", "infobrief", "safety", "safetycampaign"]:
    long_data = (
        df_wave3_parents
        .filter(regex=rf"^{f}_")
        .apply(pd.to_numeric, errors="coerce")
        .assign(role=df_wave3_parents["role"])  # Add the role column
        .melt(id_vars="role", var_name="variable", value_name="value")  # Reshape data
    )

    # Bootstrap means (1000 iterations)
    diffs = []
    for _ in range(1000):
        with warnings.catch_warnings():
            warnings.simplefilter("ignore", FutureWarning)
            sample = long_data.groupby('role', group_keys=False).apply(lambda x: x.sample(frac=1, 
replace=True))
        means = sample.groupby('role')['value'].mean()
        diffs.append(means.to_dict())

    # Get 95% CI for each group
    bootstrap_ci[f] = pd.DataFrame(diffs).quantile([0.025, 0.975]).T

# Display bootstrap CIs
for factor in ["engage", "infobrief", "safety", "safetycampaign"]:
    print(f"\n{factor.upper()} - Bootstrap 95% CI:")
    print(bootstrap_ci[factor].round(2))
```

Data wave 3 School

```
df_wave3_school = pd.read_csv(
    r"C:\Users\psipu\Dropbox\Luis\ai. Spanish Usability Paper\data_wave3_school.csv",
    sep=";",
    encoding="latin1"
)
df_wave3_school
```

Clean

```
clean_df(df_wave3_school)
df_wave3_school.head()
```

Means and standard deviations table

```
summary = (
    df_wave3_school
    .filter(regex=r"^(lesson|engage|infobrief|safetylesson|safetycontextcampaign|safetycampaign)_")
    .apply(pd.to_numeric, errors="coerce")
    .assign(role=df_wave3_school["role"])  # Add the role column
    .melt(id_vars="role", var_name="variable", value_name="value")  # Reshape data
    .assign(factor=lambda d: d["variable"].str.split("_", n=1).str[0])
    .groupby(["factor", "role"])["value"]  # Group by both factor and role
    .agg(mean="mean", sd="std") #this is the sd of all results
    .reset_index()
)
summary.round(2)
```

```
df_wave3_school["role"].value_counts()
```

```
# Bootstrap confidence intervals for mean differences
```

```
from scipy import stats
import numpy as np
import warnings

# Bootstrap confidence intervals for means
np.random.seed(42)
bootstrap_ci = {}

for f in ["lesson", "engage", "infobrief", "safetylesson", "safetycontextcampaign", "safetycampaign"]:
    long_data = (
        df_wave3_school
        .filter(regex=rf"^{f}_")
        .apply(pd.to_numeric, errors="coerce")
        .assign(role=df_wave3_school["role"])  # Add the role column
        .melt(id_vars="role", var_name="variable", value_name="value")  # Reshape data
    )

    # Bootstrap means (1000 iterations)
    diffs = []
    for _ in range(1000):
        with warnings.catch_warnings():
            warnings.simplefilter("ignore", FutureWarning)
            sample = long_data.groupby('role', group_keys=False).apply(lambda x: x.sample(frac=1,
replace=True))
        means = sample.groupby('role')['value'].mean()
        diffs.append(means.to_dict())

    # Get 95% CI for each group
    bootstrap_ci[f] = pd.DataFrame(diffs).quantile([0.025, 0.975]).T

# Display bootstrap CIs
for factor in ["lesson", "engage", "infobrief", "safetylesson", "safetycontextcampaign",
"safetycampaign"]:
    print(f"\n{factor.upper()} - Bootstrap 95% CI:")
    print(bootstrap_ci[factor].round(2))
```

Inferential tests

```
df_wave3_students.filter(regex="^engage_").apply(pd.to_numeric, errors="coerce").agg(["mean", "std"]).T
```

```
df_wave3_parents.filter(regex="^engage_").apply(pd.to_numeric, errors="coerce").agg(["mean", "std"]).T
```

```
df_wave3_school.filter(regex="^engage_").apply(pd.to_numeric, errors="coerce").agg(["mean", "std"]).T
```

Anova all matching factors

```
import pandas as pd
import statsmodels.api as sm
```

```
from statsmodels.formula.api import ols
import pingouin as pg

# ==============================================================================
# 1. DESCRIPTIVE STATISTICS: Means and SDs for each role and factor
# ==============================================================================

# Define factors and their sources (accounting for the naming issue)
all_factors = ["engage", "infobrief", "safetycampaign", "clarity", "lesson",
               "safetylesson", "safetycontextcampaign"]

descriptive_stats = []

for f in all_factors:
    combined_data = []

    # Students - NOTE: "safety" in students is actually "safetycampaign"
    if f == "safetycampaign":
        # For students, this is labeled as "safety_"
        student_data = (
            df_wave3_students
            .filter(regex=r"^safety_")
            .apply(pd.to_numeric, errors="coerce")
            .assign(role="Student")
            .melt(id_vars="role", var_name="variable", value_name="value")
        )
        if not student_data.empty:
            combined_data.append(student_data)
    elif f in ["engage", "infobrief", "clarity"]:
        student_data = (
            df_wave3_students
            .filter(regex=rf"^{f}_")
            .apply(pd.to_numeric, errors="coerce")
            .assign(role="Student")
            .melt(id_vars="role", var_name="variable", value_name="value")
        )
        if not student_data.empty:
            combined_data.append(student_data)

    # School
    if f in ["lesson", "engage", "infobrief", "safetylesson", "safetycontextcampaign", "safetycampaign"]:
        school_data = (
            df_wave3_school
            .filter(regex=rf"^{f}_")
            .apply(pd.to_numeric, errors="coerce")
            .assign(role="School")
            .melt(id_vars="role", var_name="variable", value_name="value")
        )
        if not school_data.empty:
            combined_data.append(school_data)

    # Parents
    if f in ["engage", "infobrief", "safetycampaign"]:
        parent_data = (
            df_wave3_parents
            .filter(regex=rf"^{f}_")
            .apply(pd.to_numeric, errors="coerce")
            .assign(role="Parent")
            .melt(id_vars="role", var_name="variable", value_name="value")
        )
        if not parent_data.empty:
```

```
            combined_data.append(parent_data)

    # Calculate stats if we have data
    if combined_data:
        factor_data = pd.concat(combined_data, ignore_index=True)
        stats = (
            factor_data
            .groupby("role")["value"]
            .agg(["mean", "std", "count"])
            .reset_index()
            .assign(factor=f)
        )
        descriptive_stats.append(stats)

# Combine all descriptive stats
descriptive_table = (
    pd.concat(descriptive_stats, ignore_index=True)
    .pivot(index="factor", columns="role", values=["mean", "std", "count"])
    .round(2)
)

print("\n" + "="*80)
print("DESCRIPTIVE STATISTICS: Mean (SD) and N for each Factor and Role")
print("="*80)
print(descriptive_table)

# ============================================================================
# 2. ANOVA
# ============================================================================

anova_results = {}

for f in all_factors:
    combined_data = []

    # Students - NOTE: "safety" in students is actually "safetycampaign"
    if f == "safetycampaign":
        student_data = (
            df_wave3_students
            .filter(regex=r"^safety_")
            .apply(pd.to_numeric, errors="coerce")
            .assign(role="Student")
            .melt(id_vars="role", var_name="variable", value_name="value")
        )
        if not student_data.empty:
            combined_data.append(student_data)
    elif f in ["engage", "infobrief", "clarity"]:
        student_data = (
            df_wave3_students
            .filter(regex=rf"^{f}_")
            .apply(pd.to_numeric, errors="coerce")
            .assign(role="Student")
            .melt(id_vars="role", var_name="variable", value_name="value")
        )
        if not student_data.empty:
            combined_data.append(student_data)

    # School
    if f in ["lesson", "engage", "infobrief", "safetylesson", "safetycontextcampaign", "safetycampaign"]:
        school_data = (
            df_wave3_school
```

```
            .filter(regex=rf"^{f}_")
            .apply(pd.to_numeric, errors="coerce")
            .assign(role="School")
            .melt(id_vars="role", var_name="variable", value_name="value")
        )
        if not school_data.empty:
            combined_data.append(school_data)

    # Parents
    if f in ["engage", "infobrief", "safetycampaign"]:
        parent_data = (
            df_wave3_parents
            .filter(regex=rf"^{f}_")
            .apply(pd.to_numeric, errors="coerce")
            .assign(role="Parent")
            .melt(id_vars="role", var_name="variable", value_name="value")
        )
        if not parent_data.empty:
            combined_data.append(parent_data)

    # Only run ANOVA if we have data from multiple sources
    if len(combined_data) > 1:
        factor_data = pd.concat(combined_data, ignore_index=True)

        anova_results[f] = sm.stats.anova_lm(
            ols("value ~ C(role)", data=factor_data).fit(),
            typ=3
        )

# Create formatted ANOVA table
anova_table = (
    pd.concat(anova_results, names=["factor"])
      .loc[(slice(None), "C(role)"), ["df", "F", "PR(>F)"]]
      .reset_index(level=1, drop=True)
      .rename(columns={"df": "df", "PR(>F)": "p"})
      .assign(
          df=lambda d: d["df"].astype(int),
          F=lambda d: d["F"].round(2),
          p=lambda d: d["p"].apply(lambda x: "< .001" if x < 0.001 else f"{x:.3f}".lstrip("0"))
      )
)

print("\n" + "="*80)
print("ANOVA Results:")
print("="*80)
print(anova_table)

# ==============================================================================
# 3. POST-HOC TESTS
# ==============================================================================

posthoc_results = {}

for f in all_factors:
    combined_data = []

    # Students - NOTE: "safety" in students is actually "safetycampaign"
    if f == "safetycampaign":
        student_data = (
            df_wave3_students
            .filter(regex=r"^safety_")
```

```
            .apply(pd.to_numeric, errors="coerce")
            .assign(role="Student")
            .melt(id_vars="role", var_name="variable", value_name="value")
        )
        if not student_data.empty:
            combined_data.append(student_data)
    elif f in ["engage", "infobrief", "clarity"]:
        student_data = (
            df_wave3_students
            .filter(regex=rf"^{f}_")
            .apply(pd.to_numeric, errors="coerce")
            .assign(role="Student")
            .melt(id_vars="role", var_name="variable", value_name="value")
        )
        if not student_data.empty:
            combined_data.append(student_data)

    # School
    if f in ["lesson", "engage", "infobrief", "safetylesson", "safetycontextcampaign", "safetycampaign"]:
        school_data = (
            df_wave3_school
            .filter(regex=rf"^{f}_")
            .apply(pd.to_numeric, errors="coerce")
            .assign(role="School")
            .melt(id_vars="role", var_name="variable", value_name="value")
        )
        if not school_data.empty:
            combined_data.append(school_data)

    # Parents
    if f in ["engage", "infobrief", "safetycampaign"]:
        parent_data = (
            df_wave3_parents
            .filter(regex=rf"^{f}_")
            .apply(pd.to_numeric, errors="coerce")
            .assign(role="Parent")
            .melt(id_vars="role", var_name="variable", value_name="value")
        )
        if not parent_data.empty:
            combined_data.append(parent_data)

    # Only run post-hoc if we have data from multiple sources
    if len(combined_data) > 1:
        factor_data = pd.concat(combined_data, ignore_index=True)

        posthoc_results[f] = pg.pairwise_tests(
            data=factor_data,
            dv="value",
            between="role",
            parametric=True,
            padjust="bonf",
            effsize="cohen"
        )

# Display post-hoc results
for factor, results in posthoc_results.items():
    print(f"\n{'='*80}")
    print(f"POST-HOC TESTS: {factor.upper()}")
    print('='*80)
    display(results[["A", "B", "T", "p-unc", "p-corr", "cohen"]].round(3))
```

Double-check (Brief)

```
 import pandas as pd
import statsmodels.api as sm
from statsmodels.formula.api import ols
import pingouin as pg

# Combine data
data = pd.concat([
    df_wave3_students.filter(regex=r"^infobrief_").apply(pd.to_numeric, errors="coerce").assign(role="a
Student").melt(id_vars="role", value_name="value"),
    df_wave3_school.filter(regex=r"^infobrief_").apply(pd.to_numeric, errors="coerce").assign(role="b
School").melt(id_vars="role", value_name="value"),
    df_wave3_parents.filter(regex=r"^infobrief_").apply(pd.to_numeric, errors="coerce").assign(role="c
Parent").melt(id_vars="role", value_name="value")
], ignore_index=True)

# Descriptives
print(data.groupby("role")["value"].agg(["mean", "std"]).round(2))

# ANOVA
anova = sm.stats.anova_lm(ols("value ~ C(role)", data=data).fit(), typ=3)
print("\n", anova.loc["C(role)", ["df", "F", "PR(>F)"]])

# Post-hoc
posthoc = pg.pairwise_tests(data=data, dv="value", between="role", parametric=True, padjust="bonf",
effsize="cohen")
print("\n", posthoc[["A", "B", "T", "p-unc", "p-corr", "cohen"]].round(3))
```

Double-check Ability to Engage (Safety Campaign Video)

```
 data = pd.concat([
    df_wave3_students.filter(regex=r"^safety").apply(pd.to_numeric,
errors="coerce").assign(role="Students").melt(id_vars="role", value_name="value"),
    df_wave3_school.filter(regex=r"^safetycampaign").apply(pd.to_numeric,
errors="coerce").assign(role="School").melt(id_vars="role", value_name="value"),
    df_wave3_parents.filter(regex=r"^safety_").apply(pd.to_numeric,
errors="coerce").assign(role="Parent").melt(id_vars="role", value_name="value") #attention, cause we have
two safety here; use undesrscore to avoid confusion
], ignore_index=True)

# Descriptives
print(data.groupby("role")["value"].agg(["mean", "std"]).round(2))

# ANOVA
anova = sm.stats.anova_lm(ols("value ~ C(role)", data=data).fit(), typ=3)
print("\n", anova.loc["C(role)", ["df", "F", "PR(>F)"]])
```

Double-check Safety Campaign

```
# Combine data (safetycontextcampaign_ for school, safetycampaign_ for parents)
data = pd.concat([
    df_wave3_school.filter(regex=r"^safetycontextcampaign_").apply(pd.to_numeric,
errors="coerce").assign(role="School").melt(id_vars="role", value_name="value"),
    df_wave3_parents.filter(regex=r"^safetycampaign_").apply(pd.to_numeric,
errors="coerce").assign(role="Parent").melt(id_vars="role", value_name="value")
], ignore_index=True)

# Descriptives
print(data.groupby("role")["value"].agg(["mean", "std"]).round(2))

# t-test via ANOVA (equivalent for 2 groups)
anova = sm.stats.anova_lm(ols("value ~ C(role)", data=data).fit(), typ=3)
print("\n", anova.loc["C(role)", ["df", "F", "PR(>F)"]])
```

Data Wave 2

```
df_wave2 = pd.read_csv(
    r"C:\Users\psipu\Dropbox\Luis\ai. Spanish Usability Paper\data_wave2_school.csv",
    sep=";",
    encoding="latin1"
)
df_wave2
```

Clean

```
clean_df(df_wave2)
df_wave2
```

Means and standard deviations table

```
df_wave2
```

```
tmp = (
    df_wave2
    .filter(regex=r"^instruction_")
    .replace({"na": np.nan, "nr": np.nan})
    .stack()
    .rename("raw")
    .to_frame()
    .assign(
        raw_str=lambda d: d["raw"].astype(str),
        clean=lambda d: pd.to_numeric(
            d["raw"].astype(str)
              .str.replace(",", ".", regex=False)
              .str.strip(),
            errors="coerce"
        )
    )
)

tmp.query("raw.notna() and clean.isna()")
```

```
del(tmp) #excel is treating some empy cells as something... fuck you excel.
```

```
summary = (
    df_wave2
    .filter(regex=r"^(instruction|feasability|relinstr|relbrief|relengage)_")
    .replace({"na": np.nan, "nr": np.nan})
    .replace(",", ".", regex=True)
    .apply(pd.to_numeric, errors="coerce")
    .assign(role=df_wave2["role"], id=df_wave2.index)
    .melt(id_vars=["id", "role"], var_name="variable", value_name="value")
    .assign(factor=lambda d: d["variable"].str.split("_", n=1).str[0])
    .groupby(["factor", "role"])
    .agg(
        mean=("value", "mean"),
        sd=("value", "std"),
        n_numbers=("value", "count"),
        n_participants=("id", lambda x: x[x.notna()].nunique())
    )
    .reset_index()
)

summary.round(2)
```

```
df_wave2["role"].value_counts()
```

Cronbach's alpha by factor

```
# Reliability table for wave 2
reliability_wave2 = []

factors_wave2 =
df_wave2.filter(regex=r"^(instruction|feasability|relinstr|relbrief|relengage)_").columns.str.split("_",
n=1).str[0].unique()

for factor in factors_wave2:
    cols = df_wave2.filter(regex=rf"^{factor}_").columns.tolist()

    data = (
        df_wave2[cols]
        .replace({"na": np.nan, "nr": np.nan})
        .astype(str)
        .replace(",", ".", regex=True)
        .apply(pd.to_numeric, errors="coerce")
    )

    data = data.dropna(axis=1, how="all")
    data = data.loc[:, data.nunique(dropna=True) > 1]

    if data.shape[1] < 2:
        continue

    try:
        alpha, ci = pg.cronbach_alpha(data=data)
        reliability_wave2.append({
            "factor": factor,
            "n_items": data.shape[1],
            "cronbach_alpha": alpha
        })
    except:
        pass

reliability_wave2_df = pd.DataFrame(reliability_wave2)
print(reliability_wave2_df.round(3))
```

Bootstrap confidence intervals for mean differencesm

```
# Bootstrap confidence intervals for means
np.random.seed(42)
bootstrap_ci = {}
observed_means = {}

for f in ["instruction", "feasability", "relinstr", "relbrief", "relengage"]:
    long_data = (
        df_wave2
        .filter(regex=rf"^{f}_")
        .replace({"na": np.nan, "nr": np.nan})
        .replace(",", ".", regex=True)
        .apply(pd.to_numeric, errors="coerce")
        .assign(role=df_wave2["role"])
        .melt(id_vars="role", var_name="variable", value_name="value")
    )

    # Store observed means
    observed_means[f] = long_data.groupby('role')['value'].mean()

    # Bootstrap means (1000 iterations)
    diffs = []
    for _ in range(1000):
        with warnings.catch_warnings():
            warnings.simplefilter("ignore", FutureWarning)
            sample = long_data.groupby('role', group_keys=False).apply(lambda x: x.sample(frac=1, 
replace=True))
        means = sample.groupby('role')['value'].mean()
        diffs.append(means.to_dict())

    # Get 95% CI for each group
    bootstrap_ci[f] = pd.DataFrame(diffs).quantile([0.025, 0.975]).T

# Display results with observed means and bootstrap CIs
for factor in ["instruction", "feasability", "relinstr", "relbrief", "relengage"]:
    print(f"\n{factor.upper()}")
    print(f"Observed Mean:\n{observed_means[factor].round(2)}")
    print(f"\nBootstrap 95% CI:\n{bootstrap_ci[factor].round(2)}")
```

```
! done
```
